# Increased water contamination and grow-out Pekin duck mortality when raised with water troughs compared to pin-metered water lines using a United States management system

**DOI:** 10.3382/ps/pev381

**Published:** 2016-01-14

**Authors:** A. Schenk, A. L. Porter, E. Alenciks, K. Frazier, A. A. Best, S. M. Fraley, G. S. Fraley

**Affiliations:** * Biology Department, Hope College, Holland, MI USA; # South Crossing Veterinary Center, Caledonia, MI USA

**Keywords:** angiotensin II, bacterial contamination, corticosterone, stress, water quality

## Abstract

Controversy has developed as to whether or not pin-metered water lines or water troughs are more appropriate for Pekin ducks. We hypothesized that water troughs would show improved duck body conditions and environmental quality compared to pin-metered water lines. To test this hypothesis, we housed ducks in 2 barns, one with water lines and one with water troughs. Water troughs were constructed to meet RSPCA guidelines for number and density of ducks and with recently described verandas. Ducks were divided into 4 pens per barn (*n* = 1,000 ducks/pen). The study was then repeated (*n* = 8 pens per water source) in a cross-over design so the barns each contained the opposite water source to the first experiment. We scored the ducks’ body condition using an established scoring rubric and analyzed using SAS Proc GLM-Mix as binomial data. Ducks housed with water troughs showed higher (thus worse condition; *P* < 0.001) scores for eyes, nostrils, feather quality, feather cleanliness, and foot pads. We also compared water condition, water quality, and duck mortality using a Student *t* test for both water sources each week. We found that the water troughs showed higher iron (*P* < 0.001), nitrites (*P* < 0.001), pH (*P* < 0.01), and bacterial growth (*P* < 0.001). The bacterial growth was shown to have higher (*P* < 0.001) *E. coli*, coliforms, and *Staphylococcus* in the water troughs. Water lines typically showed no bacterial growth in culture-based assays. Ducks housed with water troughs used greater (*P* = 0.001) volumes of water compared to ducks housed with water lines. Ducks with water troughs also showed a greater percent (*P* = 0.008) mortality at all ages compared to ducks with water lines. These data suggest that water troughs may not be beneficial for duck welfare and could adversely impact both environment and duck or human health.

## INTRODUCTION

The domestication of mallard ducks, *Anas platyrhynchos*, over 4,000 years ago led to the development of the Pekin duck for commercial production*.* In the wild, mallard ducks are mostly aquatic birds that obtain their food either by foraging on land or dabbling their beaks along the water's edge, in grass, or through mud (Guillemain et al., 2000; Cherry and Morris, 2008). In addition to dabbling behaviors, mallards also perform a considerable amount of preening behaviors, which allows them to remove dirt and parasites from their feathers. In a commercial setting, Pekin ducks display similar behaviors to their undomesticated ancestors, but there has been much debate over whether certain watering systems in a commercial setting adequately allow for these behaviors and the ducks’ well being.

In the United States, it is standard practice to provide poultry with a closed watering system referred to as a pin-metered water line. However, in the European Union, the use of pin-metered water lines has been criticized for not allowing ducks to perform behaviors such as dabbling, head-dipping, bathing, or swimming (for review see, (Rodenburg et al., 2005)). Previous studies suggested that pin-metered water lines were not sufficient for the welfare of ducks (Jones et al., 2009; Waitt et al., 2009; Jones and Dawkins, 2010a,b), however these studies did not investigate US management systems, and typically relied upon very small sample sizes. Other studies have also criticized water lines for causing decreased wet-preening behavior and decreased cleanliness of duck eyes and feathers (Jones and Dawkins, 2010b). Despite these many criticisms, a detailed, scientific study of watering systems has yet to be performed in the United States.

The purpose of the current study was to compare the two different styles of watering system, water troughs and pin-metered water lines, under US commercial management systems that approximate the actual number and density of ducks in a commercial barn. In order to assess the overall health of the ducks in this study, we analyzed biological, behavioral, and environmental parameters of ducks housed with the two different water sources. Given our data, it can be suggested that allowing commercial ducks access to water troughs, instead of water lines, may be a potential health risk for the ducks and for overall human food safety.

## MATERIALS AND METHODS

### Animals and Housing

In an attempt to approximate the conditions of an actual commercial barn setting, our study was conducted in two research barns owned by Maple Leaf Farms, Inc. (Leesburg, IN). Each barn was divided into 4 equal sized pens with 1,000 ducks per pen (∼0.17 m^2^ per duck). Both barns were covered with pine litter as flooring material and the only difference between the barns was the type of water source provided: pin-metered water lines or water troughs. The study ran for the duration of a typical grow-out period in the United States, approximately 34 d. After the first study was completed, the experiment was replicated in a cross-over design with the watering systems switched into the opposite barns to the first experiment, thus providing a final N = 8 pens for pin-metered water lines and N = 8 for water troughs. The ducks used in the study were a commercial Pekin strain developed by Maple Leaf Farms, Inc. Ducklings were randomly selected for both barns and placed within hours of hatch (day 0). After an initial 10-day brooding period, in approximately one-third of the pen, they were given access to the entire floor space in each pen. When the ducks reached targeted commercial weight (∼3.5 kg), at 34 days, they were all processed at the Maple Leaf Farms processing facility. The Hope College Animal Care and Use Committee approved all studies.

### Water Trough Design

**Figure 1.**
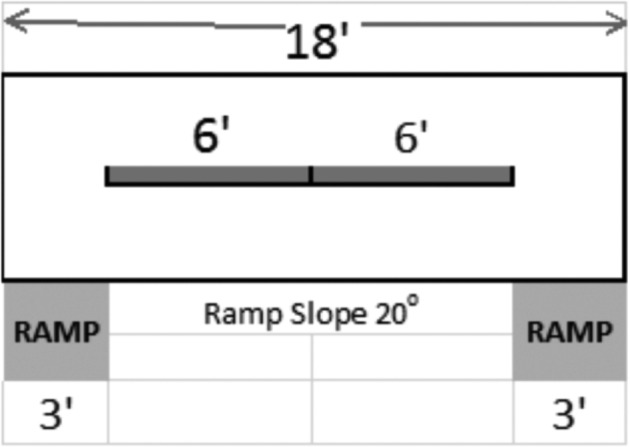
Schematic diagram of the water trough system utilized from day 14 through the end of the experiment. Water trough and veranda designed was optimized based upon published studies and RSPCA guidelines.

Water troughs were set up following the Royal Society for the Prevention of Cruelty to Animals (RSPCA) guidelines. Ducks that were 0 to 14 d old were provided with two troughs that were 8′ long by 2″ wide by 1.5″ deep. On day 15, the small water troughs were replaced with larger troughs in a veranda set-up following RSPCA guidelines. The 18-foot long veranda included 3-foot ramps, with a slope of 20 degrees, placed at each end. The two troughs that sat on top of the veranda were 6′ long by 8″ wide by 5″ deep. Figure 1 illustrates the water trough design. The troughs and the ball-cocks were emptied and wiped clean twice a day as per RSPCA guidelines (FW 2.8) and further disinfected with bleach once a week. Water was supplied to both the pin-metered lines and water troughs in a continual fashion to maintain depth of water in the troughs; the water to supply both the pin-metered water lines and water troughs came from the same well. The well was treated with chlorine in a continuous flow to maintain Cl^−^ levels between 1 to 3 ppm. In order to maintain consistency of management between barns, and to allow for minimum pine shaving usage, the amount of bedding used daily was regulated by the amount needed to cleanly cover a pen located in the barn containing a water line. In pens with pin-metered water lines, a total of 356 nipples (2.81 ducks per nipple) were arranged in 3 lines on the side of the pen in a similar placement as the troughs. Each pen had its own independent set of water lines or water troughs. Water pressure in the lines was maintained to allow for a water flow through activated pins at 60 mLs/min per pin.

### Environmental Measures

Daily, handheld instruments were used in each barn, at the center of each pen to measure carbon monoxide (CO) (Industrial Scientific T40 CO Rattler, Grainger Industrial Supply, Inc., Indianapolis, IN), ammonia (NH_3_) (TOXIRAE II, QC Supply, Schuyler, NE), and Relative Humidity (RH) (EXTECH USB Data Logger, Model RHT10, Cole Parmer, Inc., Vernon Hills, IL). The temperature was obtained in each pen of both barns using a Kestrel 3000 Wind Meter (QC Supply, Schuyler, NE).

### Water Quality Assays

#### Water Quality Analyses

A 50-mL water sample was collected one day per week of the study at several time points following the cleaning and addition of fresh water at 0600 hrs. Samples were taken by farm technicians at 15, 30, and 60 minutes and at 6 and 12 hr after 0600 hrs on the day (d 1, 9, 23, and 33) of sampling. All samples were immediately frozen to −80°C and stored until analyzed in batch. Samples were coded and analyzed in a double-blind fashion. One aliquot of each 50 mL sample was analyzed by Maple Leaf Farms Water Quality Laboratory for pH, Fe, nitrite, and nitrate levels.

#### Living Bacterial Analyses

Another aliquot of each 50 mL water sample was analyzed by Maple Leaf Farm's microbiology lab to determine the live bacterial content of the water again in a double-blind fashion. To determine live bacterial content, the number of colony forming units (**CFU**) were determined using a commercially available kit, Tempo TVC, Patterson Veterinary Supply, INc. Devens. The TVC kit and growing medium were used due to their ability to grow food microorganisms rapidly. Microorganisms were detected by the use of fluorogenic substrates. The substrates that are reduced by the microorganisms give off a fluorescent signal which is interpreted by the Tempo Reader following 40 to 48 hr of incubation at 35°C.

#### 16S rRNA Microbial Community Profiling

Further aliquots from the collected water samples at 15 minute, 6 hr, and 12 hr time points were used for DNA isolation in preparation for total microbial community analysis using the PowerLyzer PowerSoil DNA Isolation Kit (MoBio, Carlsbad, CA) according to the manufacturer's protocol, except that MP Biomedical FastPrep24 lysing matrix D tubes with 1.4 mm ceramic spheres were used in place of the MoBio glass bead tubes for sample preparation due to higher DNA yields. DNA was eluted in a final volume of 100 μL of elution buffer according to the MoBio protocol. Total community DNA was stored at −20°C.

#### Sequencing of 16S rRNA

Total community DNA samples were submitted to the Institute for Genomics and Systems Biology Next Generation Sequencing (IGSB-NGS) Core Facility at Argonne National Laboratory for sequencing of community 16S rRNA genes. Briefly, genomic DNA was amplified using the Earth Microbiome Project barcoded primer set, adapted for the Illumina MiSeq. The V4 region of the 16S rRNA gene (515F-806R) was amplified with region-specific primers that included the Illumina flowcell adapter sequences. The reverse amplification primer also contained a twelve base barcode sequence that supports pooling of up to 2,167 different samples in each lane. The final pooled library was diluted to a final concentration of 6.75 p*M* with a 10% PhiX spike for sequencing on the Illumina MiSeq (Caporaso et al., 2012).

#### Microbial Community Data Analyses

The Quantitative Insights into Microbial Ecology (QIIME) software package, version 1.8.0 (Caporaso et al., 2010b) was used to analyze 16S microbial sequencing data. We utilized custom shell scripts to perform “upstream” and “downstream” processing stages as recently described (Navas-Molina et al., 2013). All steps requiring comparison of sequences to a reference database used the GreenGenes database, release 13_8 (DeSantis et al., 2006). For the upstream analysis steps, we performed demultiplexing and quality-filtering for Illumina based sequence reads using default values. Clustering of sequencing reads that passed quality filters into operational taxonomic units (**OTU**) was performed through an open-reference strategy at a threshold of 97% identity, using uclust (Edgar, 2010). Taxonomic assignment of representative OTU was performed using the QIIME rtax workflow in order to take advantage of paired end sequencing reads (Soergel et al., 2012). The rtax settings allowed for inclusion of OTU identified by non-paired reads (–single ok option). Chimeric sequences were removed using ChimeraSlayer (Haas et al., 2011). In order to construct a phylogenetic tree of the identified OTU, sequences were aligned using PyNAST (Caporaso et al., 2010a) against the GreenGenes core set template (DeSantis et al., 2006; McDonald et al., 2012). A phylogenetic tree was constructed using FastTree 2 (Price et al., 2010) within the QIIME workflow. Finally, an OTU table in BIOM format (McDonald et al., 2012) was produced, along with a complete metadata mapping file for use in downstream analysis steps.

We performed secondary filtering of OTU to minimize the effect of very low abundance OTU, using the recommended value of <0.005% of the total number of sequences (Bokulich et al., 2013) as the threshold for removal of OTU from further consideration. The filtered BIOM table, phylogenetic tree and metadata sample table were passed to the core diversity analysis workflow (Lozupone and Knight, 2005; Navas-Molina et al., 2013) in QIIME to perform initial taxa summarization, alpha diversity, beta diversity, and taxon differential distribution analyses. Samples were rarefied to 9,700 OTU based on the distribution of samples after BIOM table filtering. A jackknifed beta diversity analysis (Lozupone et al., 2011) was conducted to assess statistical variation of sample location in principle coordinate analysis (PCoA) plots. We used EMPeror (Vázquez-Baeza et al., 2013) to visualize PCoA plots. Following initial evaluation of the data, the BIOM table was variously filtered to focus on particular sample comparisons, specifically a comparison of week 1 and week 5 water samples at beginning and end of day sampling time points; analyses for this subset were rarefied at 9,700 OTU. A 2-way analysis of variance (ANOVA) was used to test for significant differences between observed numbers of bacterial species in targeted comparisons. Sequencing data have been deposited in association with the project PRJEB11269 (http://www.ebi.ac.uk/ena/data/view/PRJEB11269) in the European Nucleotide Archive (ENA).

### Duck Biological Assays

The biological conditions of the ducks were assessed through body condition scoring, analyses of gut microbial ecology, and serum hormone levels for corticosterone (**CS**) and angiotensin II (**AgII**). Body condition scoring was performed at ages 9, 23, and 33 d using a published rubric (Fraley et al., 2013; Karcher et al., 2013; Colton and Fraley, 2014). At the 3 specified ages, researchers used gates to randomly select 25 ducks in four corners of each pen, resulting in a total of 100 ducks/pen. Each duck was scored on eyes, nostrils, foot pads, feather quality, and feather cleanliness according to the rubric previously mentioned. The scores were averaged among the 100 ducks in each pen giving a final N = 8 per water treatment.

Gut ecology samples were obtained on d 5, 23 and 33. In each pen, 3 apparently healthy ducks (*n* = 12 per water treatment) were selected at random and immediately euthanized using Fatal Plus (400 mg/kg pentobarbital, IP, Patterson Veterinary Supply, INc. Devens). Pentobarbital is a well-known inhibitor of gastrointestinal motility and hypothalamic neurosecretion. The ducks were weighed and the contents of their paired caeca were collected for gut microbial ecology assay using next generation sequencing as described above.

On d 9, 23, and 33 we also collected blood serum to determine circulating levels of CS as an indirect measure of stress. We also analyzed serum AgII levels as a biological indicator of hydration and/or thirst. Serum levels of CS in ducks were measured by ether extraction and radioimmunoassay (**RIA**) at the Endocrine Technology and Support Core Lab (ETSC) at the Oregon National Primate Research Center/Oregon Health & Science University (Beaverton, OR). Briefly, samples (4 to 20 μL) for CS were extracted in 5 mL of ether in 13 × 100 glass tubes (previously baked at 500°C for 30 min), dried under forced air, and analyzed by specific CS RIA. Hormonal values were corrected for extraction losses determined by radioactive trace recovery at the same time with sample extraction; hot recovery usually was better than 90%. The sensitivity was 5-pg/tube for the CS RIA. All samples were analyzed in one assay with intra-assay variation of 6.3% based on an ETSC serum pool as internal controls (N = 8). The value of the ETSC serum pool in this assay was within 10% of previous CS assays (N = 20).

Plasma levels of angiotensin II (AII) in ducks were measured by methanol extraction and radioimmunoassay as instructed by the manufacturer (Alpco, Salem, NH). Briefly, samples (75 to 125 mL) were extracted in 2 mL of methanol in 13 × 100 glass tubes (baked at 500°C for 30 min), dried under forced air and analyzed by specific AII RIA. Hormonal values were corrected for extraction losses determined by radioactive trace recovery at the same time with sample extraction; hot recovery usually was better than 90%. The sensitivity was 2 pg/mL for the AII RIA. All samples were analyzed in one assay with intra-assay variation of 4.3% based on supplied internal controls. The values for the 3 ducks in each pen were averaged giving a final N = 8 per water treatment.

### Production Data

The feed to weight gain ratio was assessed by weighing 100 ducks/pen that were apparently healthy and randomly selected each week. The feed intake of these ducks was assumed to be representative of that of the entire pen. The mortality rates were recorded and all ducks were necropsied by technicians employed by Maple Leaf Farms, Inc. to estimate cause of death. We also obtained carcass condemnation numbers from US Department of Agriculture inspectors who were unaware of the treatment conditions used in this study at the processing plant (Maple Leaf Farms, Inc., Milford, IN). Feed conversion ratio (**FCR**) was defined as total feed consumed/live weight gain. Since no adjustments were made for mortality to determine FCR, we then calculated the feed efficiency index (**FEI** = ((liveability × live weight) /(age × FCR)) × 100). Livability is 1 minus the mortality ratio to all ducks placed on day 1 and is usually ranging from 0.95 to 0.85 (5 to 15% mortality).

### Statistical Analyses

The body scoring data were analyzed using SAS Proc GLM-Mix as binomial data. Data were organized by characteristic with total number of a score divided by total number of observations (100) per pen and age. Model was the response variable explained by age and water source type and their interaction. The production and environmental data were analyzed using Proc Mixed with the same model as described above. The environmental and hormonal data were normally distributed and analyzed using an ANOVA with Fisher's PLSD as the post hoc test. A probability less than 0.05 was considered to be significant.

## RESULTS

### Barn Environment

#### Barn Temperature, Humidity, and NH_3_ Levels

**Figure 2.**
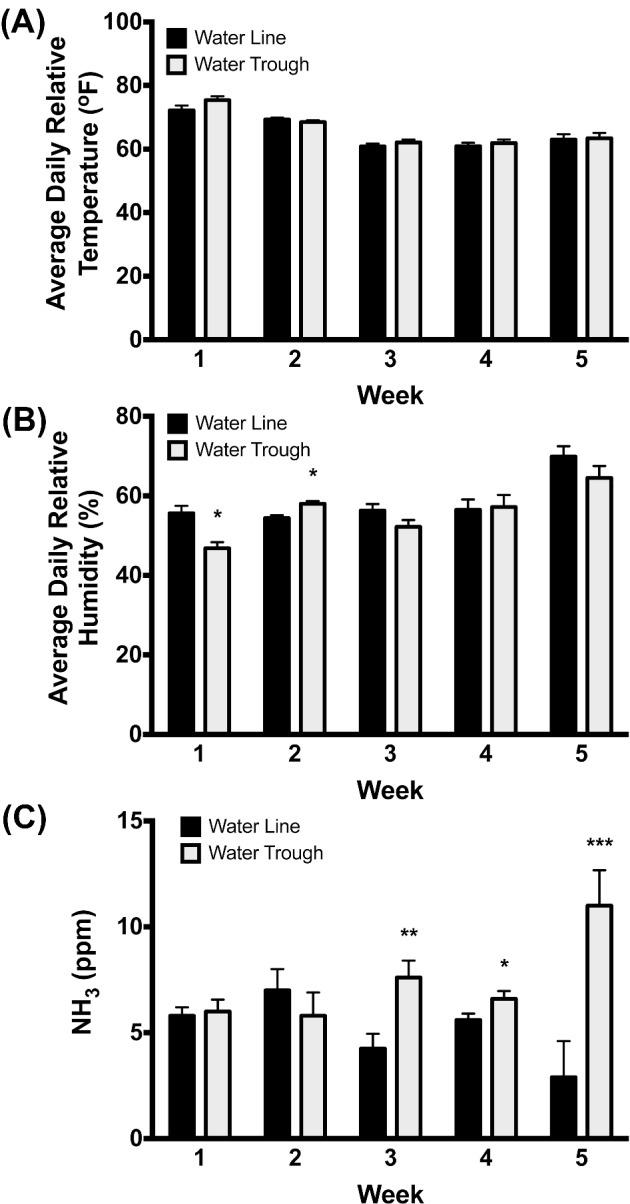
Barn environmental conditions. (A) No differences were observed in the average daily temperature among barns. (B) Minimal differences were observed in humidity levels in barns with water troughs compared to pin-metered water lines. (C) Barns with water troughs showed significantly increased NH_3_ levels than barns with pin-metered water lines during the last 3 wk of the study. * = *P* < 0.05, ** = *P* < 0.01, *** = *P* < 0.001.

No differences in NH_3_ levels were observed in barns with either water source during the first 2 weeks (wk) of the study. However, the barn with water troughs did have increased NH_3_ levels when averaged over each week over the last 3 wk of the study, thus wk ending on d 21 (*P* = 0.008), day 28 (*P* = 0.032), and day 33 (*P* = 0.001). There were no differences in the daily temperatures between the barns at any time of the day, each day throughout the experiment. However, the humidity in the water trough barn was slightly lower than the water line treatment on day 7 (*P* = 0.038) and slightly higher than the water line treatment on day 14 (*P* = 0.043), but no other differences were observed in humidity levels between barns with either water source. Figure 2 illustrates these data.

#### Water Use

**Figure 3.**
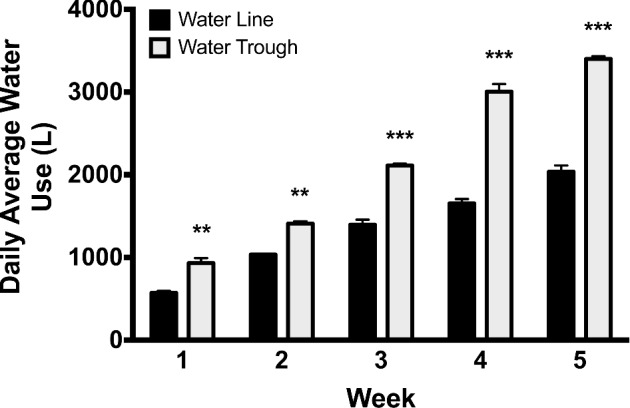
Water usage. Daily water use was averaged for each 7 day (week) time period of the study. During every week of the study, barns with water troughs showed significantly great water use compared to barns with pin-metered water lines. ** = *P* < 0.01, *** = *P* < 0.001.

At every time point measured during the experiment (daily) the barns with water troughs used a considerably greater volume of water compared to the barns with water lines. During week 1 and week 2, ducks provided with water troughs used more (*P* = 0.009) water than the ducks provided with water lines. By wk 3, 4, and 5, the difference in water usage increased (*P* < 0.001) where ducks housed with water troughs again recorded more water usage than those ducks provided with pin-metered water lines (Figure 3).

#### Water pH, Iron, and Nitrite Levels

**Figure 4.**
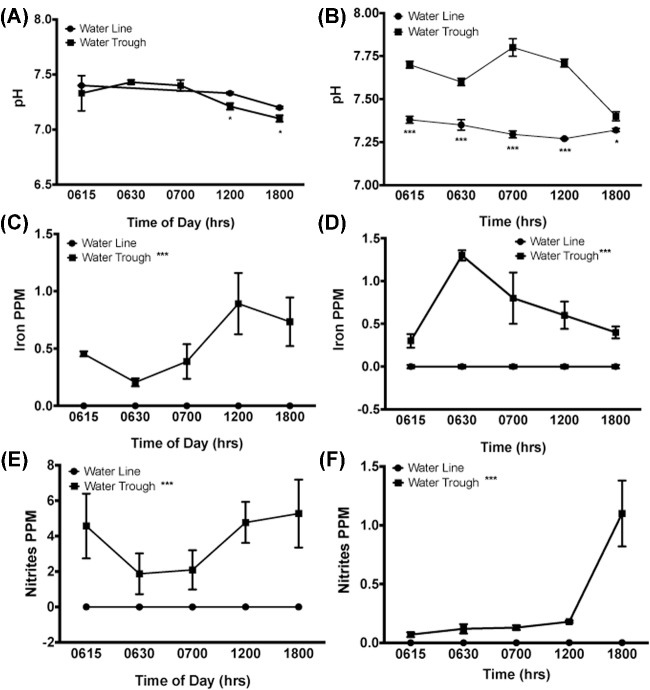
Water quality. During the first week (A) water pH was significantly different but not until 6 and 12 hr after cleaning of the water troughs, presumably due to the fact that the troughs were much smaller and more difficult for the ducks to enter. However, by week 5 (B) water pH was significantly increased in the water trough at all time points sampled. Water Fe levels were significantly increased at all time points assayed during week 1 (C) and week 5 (D), presumably due to dropped feed and feces into the water. Similarly, nitrites were also raised at all time points assay at both week 1 (E) and 5 (F) again likely due to feed contamination. *** = *P* < 0.001.

Higher pH levels were observed on day 9 for water lines at 1200 hr and 1800 hr. By day 33, the pH levels were again observed to be different, with the water troughs showing higher pH levels recorded at 0615 hr (*P* < 0.001), 1200 hr (*P* < 0.001), and 1800 hr (*P* = 0.029). The water source for the entire study came from well water with undetectable iron, nitrate, and nitrite levels. For every day that water quality was determined for each water source, the water within the troughs had higher (*P* < 0.001) levels of iron and nitrites beginning at 15 minutes after the water source was cleaned out and refilled each day. This trend continued for the rest of the day compared to water in the water lines. No differences in nitrates were observed between water samples from the pin-metered water lines and water troughs (data not shown). Figure 4 illustrates representative water quality pH, iron and nitrite levels during wk 1 and 5.

#### Microbiology

**Figure 5.**
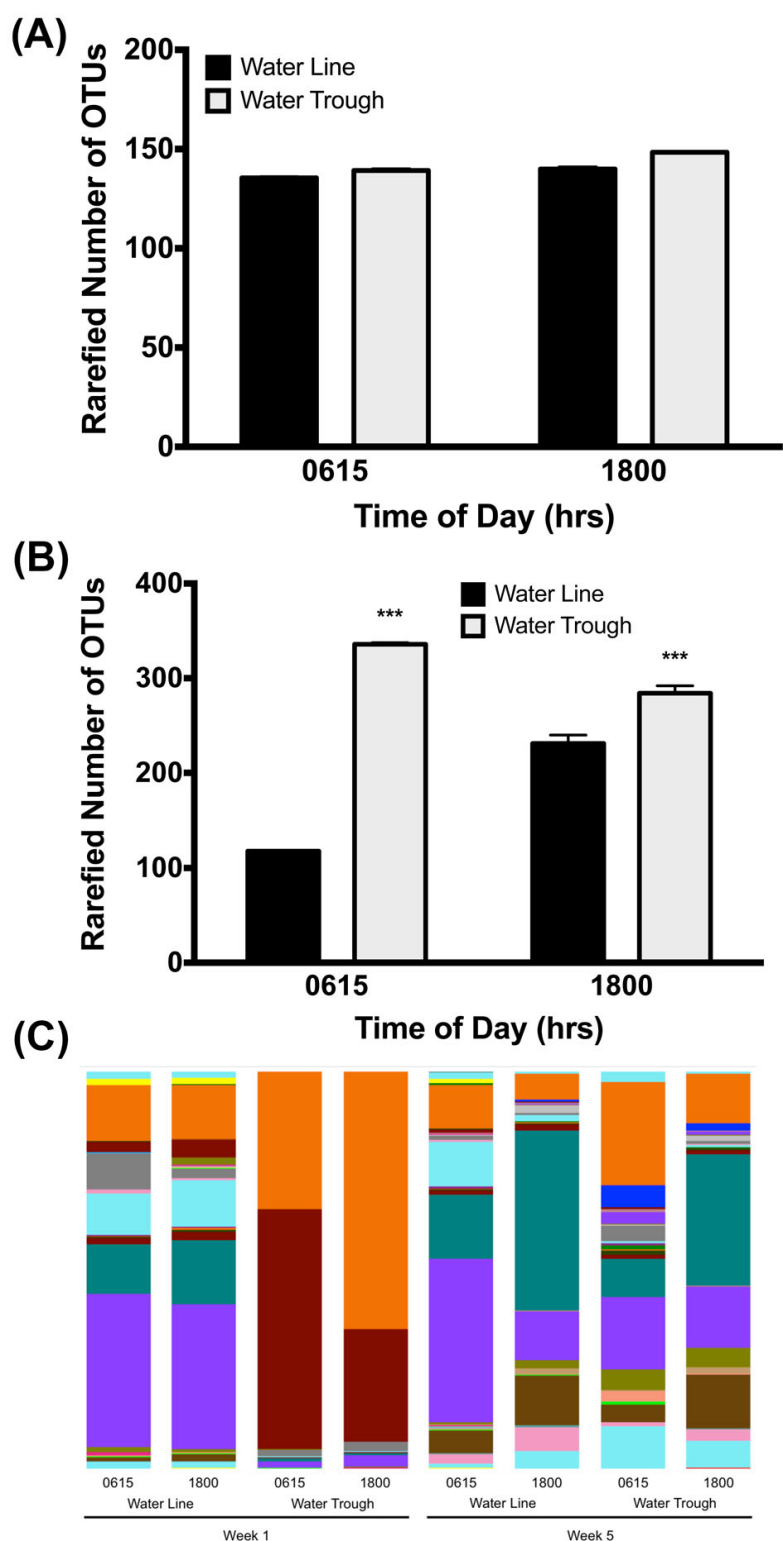
Bacterial content of water. Microbial community profiling data were used to estimate the number of unique bacterial taxa observed in each sample. The number of observed species at an even sampling depth of 9,700 sequencing reads was used for comparisons in panels A and B. The sampling depth was shown to be sufficient to cover the diversity of the community based on alpha diversity measures as implemented in Qiime 1.8. Statistical significance of differences between number of species observed was determined by two-way ANOVA. (A) An analysis of samples from week 1 of the grow out period showed no significant differences between the number of observed OTU from different water sources or different sampling times after daily cleaning. (B) An analysis of samples from week 5 showed significant differences between the number of observed OTU in water samples with respect to time of day sampled ((F1, 156) = 26.44, *P* < 0.0001) and water source ((F1, 156) = 509.4, *P* < 0.0001). Specifically, sampling times late in the day had more observed OTU than sampling within half an hr of cleaning, and water troughs had more observed OTU than water lines. (C) The relative abundance of bacterial taxa for the Order level are shown for water samples from pin-metered lines, troughs, early and late day sampling times, and wk 1 and 5 of the grow out period. Distinct differences in taxa observed and abundances are apparent for the samples at different times of day and in different weeks.

Water samples collected from week 1 and week 5 of the grow out period contained an average of 100 to over 300 distinct, bacterial taxa based on 16S community profiling. Samples obtained from water lines and water troughs early in the morning (0615 hr) and late in the day (1800 hr) might be expected to be different with respect to the number of OTU observed, given the increased time from cleaning of lines and troughs to sampling. During week 1, there is not an increase in the number of OTU observed at early and late time points for water lines or water troughs (Figure 5A). In contrast, by week 5 of the grow out period there is a significant increase (*P* < 0.001) in the number of OTU observed early and late in the day (Figure 5B). This is true for both water line and water trough samples. Further, there is a difference (*P* < 0.001) in the number of OTU observed in week 5 between water line and water trough samples, indicating that water troughs increase exposure of ducks to more bacterial taxa.

While approximately the same number of OTU is seen in samples from water lines and water troughs during week 1, it is important to note that the kinds of bacteria present in the samples are very different. This is evident in an analysis of the relative abundance of the bacterial OTU in a sample (Figure 5C; histogram key is presented in Supplemental Figure S1). Water line samples have a greater diversity of high-level taxonomic groups (Order level depicted), whereas two groups within the class Gammaproteobacteria (Enterobacteriales and Pseudomonadales) dominate water trough samples. The diversity of OTU in water trough samples has shifted dramatically by week 5, more closely resembling all other water samples. However, distinct differences in OTU abundance and presence are evident.

**Figure 6.**
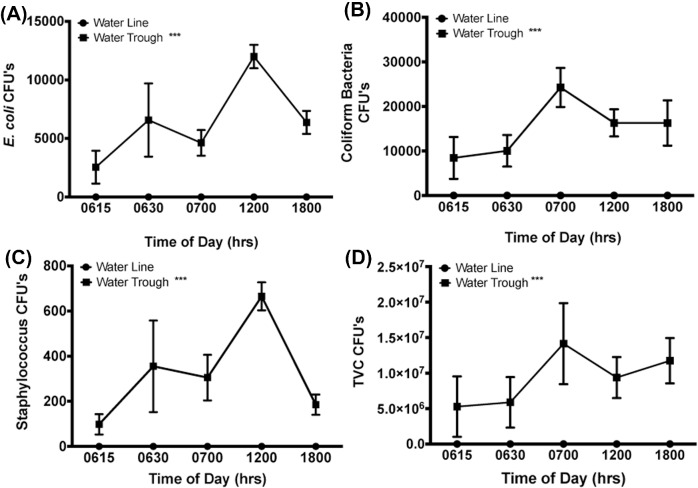
Analyses of living bacteria in water samples. A representative illustration of the number of colony forming units (CFU) during the last week of the study (day 33) for (A) *E. coli*, (B) all coliform bacteria, (C) staphylococci, and (D) the total viable bacteria (TVC). At all time points, water samples from troughs showed significantly increased number of culturable bacteria than samples from pin-metered water lines. *** = *P* < 0.001.

Analyses of the number of CFU in the water samples taken from pin-metered water lines and water troughs also illustrated considerable differences throughout the experiment. Water samples from the water troughs at each week and all time points within 15 minutes following the clean-out of the water troughs at 0600 hrs showed higher levels (*P* < 0.001) of *E. coli*, coliform bacteria (*P* = 0.00062), *Staphylococcus* genus (*P* = 0.003), and total number of culturable bacteria (**TVC**; *P* < 0.001) than water line samples which contained no culturable bacteria under these conditions. Figure 6 illustrates representative data from these analyses from sample collection on day 33.

### Duck Biological Measures

**Figure 7.**
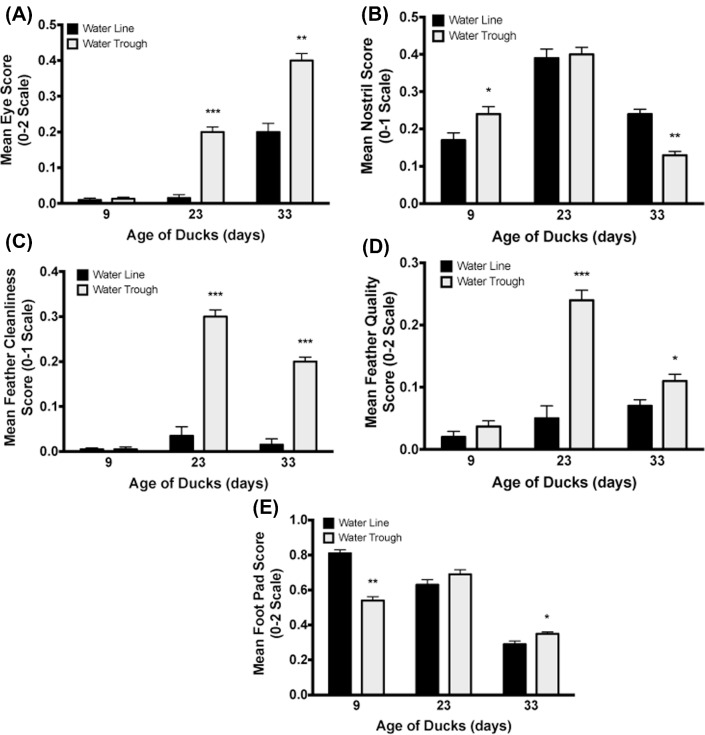
Duck body condition scoring. Ducks raised with water troughs showed significantly increased (and thus poorer quality) eye (A), feather cleanliness (C), and feather quality (D) scores compared to ducks raised with pin-metered water lines at ages 23 and 33 d. (B) Ducks raised with water troughs showed significantly higher nostril scores at age 9 d, but significantly lower nostril scores at age 33 d compared to ducks raised with pin-metered water lines. (E) Ducks raised with water troughs showed significantly lower foot pad scores at 9 d of age, but then significantly higher scores at 33 d of age compared to pin-metered water lines. * = *P* < 0.05, ** = *P* < 0.01, *** = *P* < 0.001.

There were no differences in body weight observed at any age between the treatment groups (data not shown). Ducks did not show any differences in eye, feather cleanliness, or feather quality scores between treatment groups on day 9. However on day 9, ducks raised with water troughs did have slightly higher (*P* = 0.037) mean nostril scores (thus worse condition) and lower footpad scores than the ducks with water lines. By day 23, ducks with water troughs had higher (thus worse) mean eye (*P* < 0.001), feather cleanliness (*P* < 0.001), and feather quality scores (*P* < 0.001) than ducks with water lines; no differences in nostril or footpad scores were observed. This trend continued until the end of the experiment where on day 33, ducks housed with water troughs had higher mean eye and nostril scores (*P* = 0.007), higher feather cleanliness scores (*P* < 0.001), and higher mean feather quality scores (*P* = 0.041) compared to ducks housed with water lines. Figure 7 illustrates the duck body condition scores.

#### Plasma Hormone Levels

**Figure 8.**
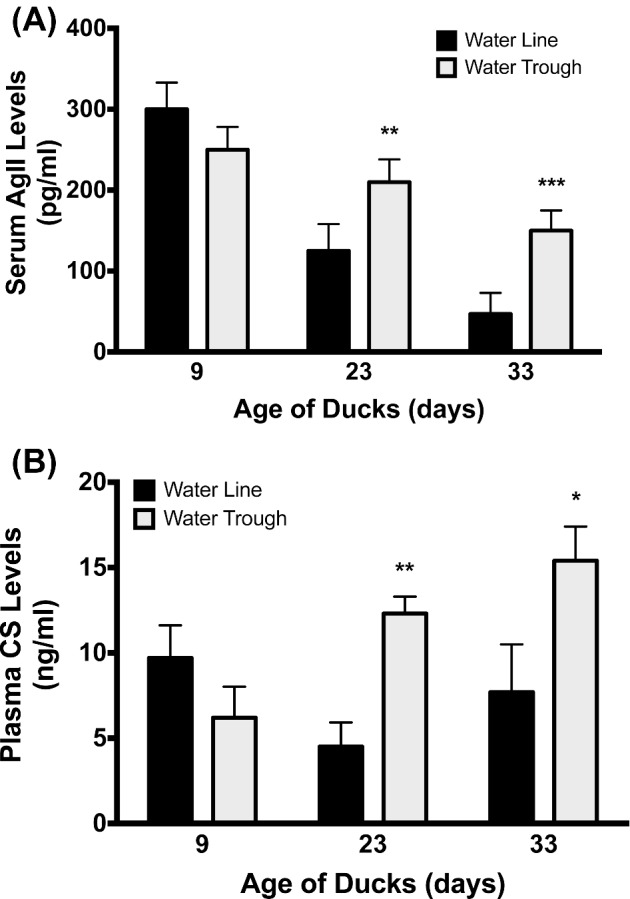
Hormone analyses. (A) At 23 and 33 d of age, ducks showed significantly increased levels of circulating angiotensin II (AgII) and (B) corticosterone (CS) levels compared to ducks raised on pin-metered water lines suggesting negative osmotic homeostasis and stress, respectively. * = *P* < 0.05, ** = *P* < 0.01, *** = *P* < 0.001.

On day 9 no differences were observed in plasma CS or AGII levels in ducks with either water source. However, on day 23 ducks housed with water troughs had higher plasma levels of both plasma CS (*P* = 0.008) and AGII (*P* = 0.007) compared to ducks housed with water lines. On day 33, ducks with water troughs still showed higher levels of CS (*P* = 0.03) and AGII (*P* < 0.001) compared to ducks housed with water lines. These data are illustrated in Figure 8.

#### Cecal Microbiome

**Figure 9.**
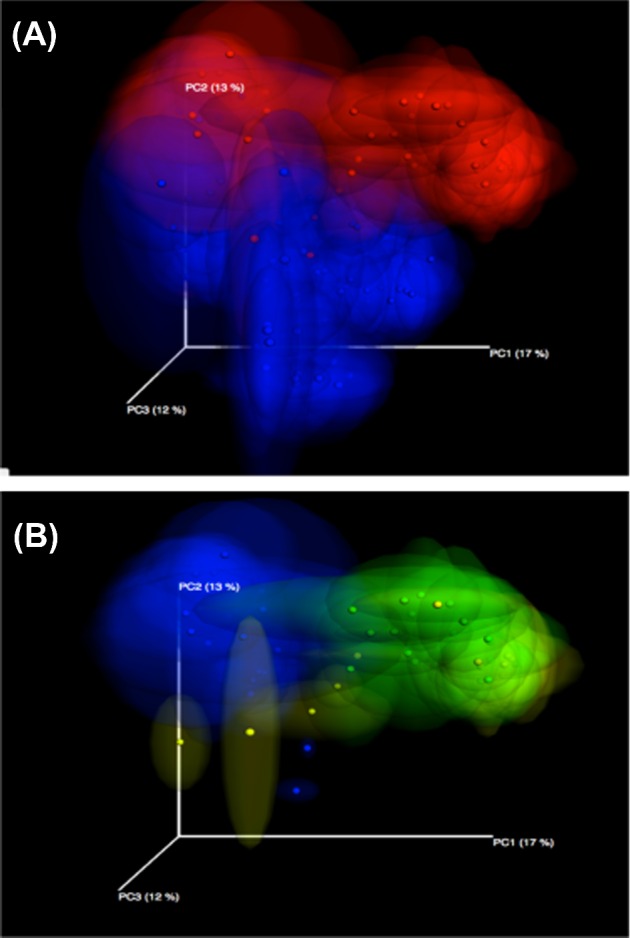
Microbial community profiling of duck cecal contents and water. Principal coordinate analyses (PCoA) of duck microbial community profiles are shown. Each sample is plotted as a point in multi-dimensional space, where the distance between points is the difference in the bacterial community profiles for each sample. A jackknife statistical resampling approach was used to show the area in which a single point could be plotted, giving a measure of confidence in the location of each point. (A) Samples from duck cecal contents (red) and water (blue) show distinct bacterial community profiles, indicating that the sources of bacteria in the water samples include, but are not limited to, duck fecal matter. (B) Duck cecal samples are colored by age of the duck (day 5, blue; day 23, green; day 33, yellow). There is a clear developmental shift in the bacterial community profiles as the ducks mature during the grow-out period.

In addition to water samples, duck cecal contents were sampled on selected d through the grow out period and bacterial community profiling was performed. An analysis of the pairwise differences between bacterial taxa present in each sample show that OTU profiles found in the duck cecal samples are distinctly different than the OTU profiles found in water samples (Figure 9A). This shows that while ducks are certainly contributing to OTU observed in water samples, other environmental sources of bacteria are influencing the bacteria present in the open water of the troughs. Focusing solely on the duck samples, there is a clear change in the bacterial communities as the ducks age (Figure 9B), though this remains distinct from water sample community profiles. There were no differences in the cecal bacterial communities of ducks raised with water troughs compared to those ducks raised with water lines (data not shown).

### Duck Production

#### Food Intake

**Figure 10.**
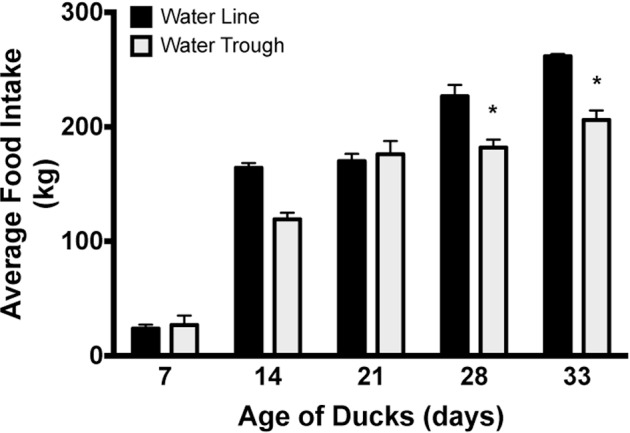
Food intake. No differences were observed in the average daily food intake at ages 7, 14, or 21 d of age, however ducks raised with water troughs appeared to eat significantly less feed at ages 28 and 33 d. These differences are most likely due to the reduced number of ducks in the barns with water troughs compared to barns with pin-metered water lines at the later ages. * = *P* < 0.05.

Ducks housed in water troughs had lower average feed intake than those housed with water lines on d 28 and 33 (*P* = 0.03 and *P* = 0.024, respectively; Figure 10).

#### FCR/FEI

**Figure 11.**
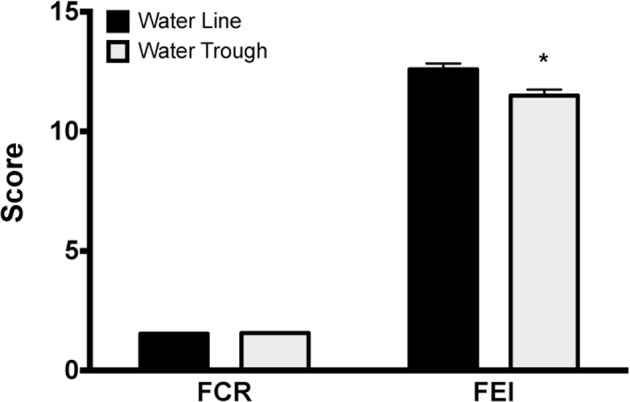
Production data. Although no differences were observed among barns in the feed conversion ratio (FCR), ducks raised with water troughs showed significantly reduced feed efficiency ratio compared to ducks raised with pin-metered water lines. * = *P* < 0.05.

The FCR was not different between ducks raised on water troughs and ducks raised on water lines. However, the FEI was lower (*P* = 0.046) for ducks raised on water troughs compared to ducks raised on water lines (Figure 11).

#### Mortality

**Figure 12.**
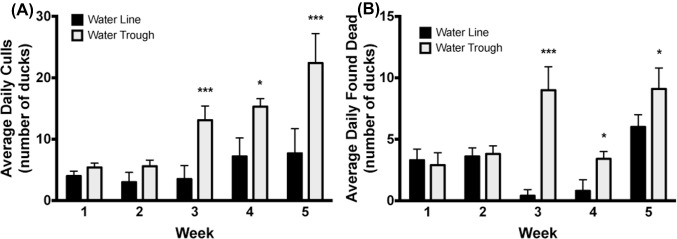
Duck mortality. Ducks raised with water troughs showed significantly increased number of culls (A) and found dead (B) compared to ducks raised with pin-metered water lines due to lameness or pathological conditions. * = *P* < 0.05, *** = *P* < 0.001.

There was no difference in duck mortality for wk 1 and 2, but the average total mortality for ducks housed in water troughs was higher at wk 3, 4, and 5 (*P* < 0.001, *P* = 0.04, *P* < 0.001, respectively). Similarly, the water trough treatment group had a greater average number of ducks found dead on wk 3, 4, and 5 (*P* < 0.001, *P* = 0.04, *P* = 0.04, respectively) and there were more ducks culled in the water trough treatment group for wk 3, 4, and 5 (*P* < 0.001, *P* = 0.03, *P* < *P* < 0.001, respectively). Overall, the percent total mortality was higher for ducks raised with water troughs compared to those housed with water lines (*P* = 0.008; Figure 12).

#### Condemns

**Figure 13.**
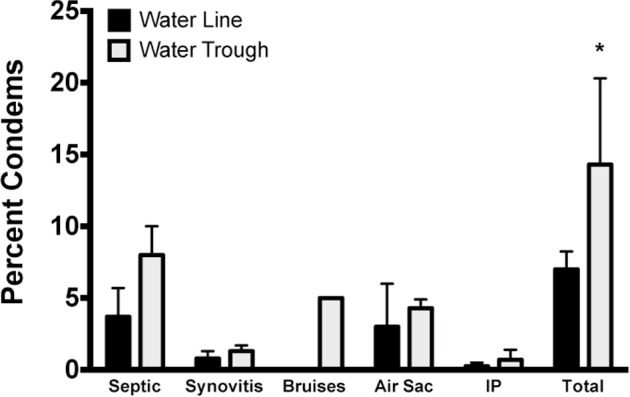
Condemned duck carcasses. Ducks raised with water troughs showed a significantly increased number of condemnations at the processing plant by inspectors unaware of the treatments. * = *P* < 0.05.

There were no differences observed between water sources for the number of ducks condemned individually for septic, synovitis, bruises, air sacs, or infection peritonitis (**IP**). However, there was a difference in the total number of ducks condemned overall. Ducks raised in the water trough treatment group had a larger (*P* = 0.046; Figure 13) number of ducks condemned.

## DISCUSSION

The purpose of this study was to test the hypothesis posited by others (Jones et al., 2009; Waitt et al., 2009; O'Driscoll and Broom, 2011) that water trough systems improve duck health and welfare compared to pin-metered water lines. In order to assess the welfare of the ducks, we housed hatchlings in barns containing either a pin-metered water line (US standard) or a water trough with a veranda (UK standard design) until the grow-out age of 34 d. We compared biological, behavioral, and environmental parameters for the ducks with the two different water sources. Overall, our data indicated that water troughs utilized a considerably greater volume of water than pin-metered lines. Compared to pin-metered lines, water troughs also had higher levels of bacterial contamination, and concurrently the ducks showed increased (thus poorer) body condition scores. Ducks raised with water troughs resulted in increased duck mortality and plant condemnations by the USDA inspectors. Overall, we found that ducks housed with the open water troughs were of much poorer quality and had a much higher mortality rate, possibly due to a high degree of water contamination. From an economic standpoint, there was a much greater amount of water used by ducks housed with water troughs compared to the pin-metered water lines. Thus, we conclude that the hypothesis must be rejected. This is the first comprehensive study to compare the two watering systems following US management practices that includes the use of pine shavings; however, our data do differ from opinions reported elsewhere (Jones et al., 2009; Waitt et al., 2009; Jones and Dawkins, 2010a,b) that water lines are inadequate for duck welfare, as determined by body condition and behavioral scoring.

We utilized an established body condition scoring rubric to assess eye, nostril, feather quality/cleanliness, and footpad health for ducks housed with each of the watering systems (Fraley et al., 2013; Karcher et al., 2013). A previous study (Waitt et al., 2009) compared body conditions for Pekin ducks that were provided access to 4 different types of watering systems. That study concluded that the limited amount of water provided by a water line was inadequate and kept ducks from performing preening activity; however, the nipple system they utilized only contained 4 nipples for all the ducks in the study (up to 24 ducks per nipple). Also, there was no mention in that study (or others) in regards to the flow rate of water through the pin-metered water lines. The water line management protocol utilized in the Waitt et al. study (Waitt et al., 2009) is considered inadequate under US management systems. In our current study, we utilized similar nipple numbers as in industry allowing less than 3 ducks per nipple. The Waitt et al. (2009) findings contradicted original studies reviewed by (Rodenburg et al., 2005) that demonstrated that wet preening did in fact occur with pin-metered water line systems. The purpose of preening is to maintain feather quality and cleanliness in healthy birds. Our laboratory has examined and published observations of nearly 1,000,000 ducks raised with pin-metered water line systems; ducks observed in all our publications showed excellent body condition, particularly with eye and feather quality, and feather cleanliness regardless of other differences in management or environmental conditions (Fraley et al., 2013; Karcher et al., 2013; Campbell et al., 2014; Colton and Fraley, 2014; Rice et al., 2014; Campbell et al., 2015). Thus, we have not observed any consistent or specific evidence showing that pin-metered water lines are unable to support adequate preening for Pekin ducks. In point of fact, a recent study showed that commercially housed ducks preen equally as often regardless of where they are in relation to the water source within a barn (Rice et al., 2014). We did not quantify preening behavior in our current study, however, even if ducks with water troughs did preen more frequently than ducks raised with pin-metered water lines they did so with dirty, contaminated water.

The water that was used to supply both the water lines and the water troughs came from the same well and were equally treated with chlorine. Despite the attempts to keep the trough water clean, within 15 minutes of cleaning the troughs and fresh water being added each day, the water in the water troughs showed significantly increased levels of culturable bacteria and significantly increased numbers of bacterial taxa within the water. In contrast, although bacteria that were potentially killed by chlorine-treatment of the water and could still be detected by next generation sequencing, the water samples from the water lines always showed zero CFU regardless of week or time of day. As the day progressed, water conditions in the troughs worsened. The water troughs contained water with increasing pH, iron, and nitrite levels. It is likely that these contaminants are due to feed and feces being dropped into the troughs by the ducks. While it is not clear from these data what the relationship is between increased bacterial counts from culture-based food quality monitoring tests and specific bacterial taxa identified in microbial community profiles based on sequencing methods, it is plausible that the health of ducks and farm workers could be at higher risk in later periods of the grow-out period as bacterial loads increase and as particular bacterial taxa change in abundance. This is consistent with data on the number of ducks culled or found dead during this study.

Ducks raised on water troughs had reduced FEI compared to ducks with water lines. The reduced FEI was observed despite similarities in FCR between treatment groups. These observations can be explained by a very large number of ducks found dead or culled each day with water troughs compared to the barns with pin-metered water lines. The ducks that were culled were typically due to lameness because of swollen, inflamed joints, or pathologies such as severely infected eyes or excessive ascites. Since the ducks that were found dead each morning could have died overnight, prior to 0600 hrs, it was not possible to determine the specific cause of death. The ducks that were found dead were never found within the water trough; therefore, contamination of the water was most likely not caused by a duck fatality within the water. It is therefore reasonable to hypothesize that increased water trough contamination from opportunistic, pathological bacteria led to the observed increase in disease and lameness. Other studies that compared water troughs to pin-metered water lines did not report the number of culled ducks nor the mortality, so it is not clear how results from this study compare to previous reports (Heyn et al., 2006; Briese et al., 2009; Heyn et al., 2009; Jones et al., 2009; Waitt et al., 2009; O'Driscoll and Broom, 2011). The conditions for ducks to spread disease in open water has been well established in both commercial and natural water systems (Wolf and Burke, 1982; Kaleta et al., 1983; Spieker et al., 1996; Pearson and Cassidy, 1997; Hansen et al., 2000; Loudon et al., 2011; Green et al., 2012; Lebarbenchon et al., 2012; Dong et al., 2013; Kobayashi et al., 2013; Wozniakowski and Samorek-Salamonowicz, 2014). In our study, the increased incidence of diseased ducks and the increased water contamination in the water troughs is not only an obvious health hazard to the ducks themselves, but it also presents dangers for humans who work in the commercial duck industry as has been demonstrated with other food animal species (Huijsdens et al., 2006; Khanna et al., 2008; Golding et al., 2010; Oppliger et al., 2012; Boost et al., 2013; Osadebe et al., 2013; van der Mee-Marquet et al., 2014; van Cleef et al., 2015) and further, water contamination could lead to food safety issues.

Our study set out to analyze how two water systems influence a duck's welfare from hatch to grow out. Even though water troughs seem like a natural water source due to the mallard duck ancestry of the Pekin, the open water systems may not be appropriate for the domesticated and barn-housed Pekin ducks. Natural water systems such as ponds, lakes, and rivers, have a vastly greater volume of water per duck than is possible in a commercial system. Furthermore, natural water systems contain many grasses, plankton, fishes, and invertebrates that all act as a natural filtration system for the water, and the ducks are simply another part of this natural ecosystem. Obviously, in a commercial barn setting, this complete ecosystem is missing. These observations further bolster the opinion that open water systems for Pekin ducks within US management practices are inappropriate. Compared to the ducks housed with pin-metered water lines, the ducks housed with water troughs had more incidences of poorer body condition scores and greater overall mortality. For this reason, we must reject the hypothesis that water troughs are better for duck welfare than pin-metered water lines. By housing ducks with water troughs, the ducks are potentially at an increased risk for disease and death due to the vast amounts of bacteria that accumulate each day within open water. A recently described new cup-style drinker (Bal, 2014) claims to improve water quality issues, however these claims were not compared to other drinking systems, nor have the claims been confirmed scientifically. Regardless, many studies reviewed by Rodenburg (Rodenburg et al., 2005) that compared pin-metered water lines to water troughs raise serious concerns about open water systems and water contamination. In fact, a recent study that compared different styles of water troughs stated that water troughs could negatively impact long-term production and public health (Liste et al., 2013). Our study has further bolstered the opinion that open water systems in Pekin duck barns act as a “sink” for environmental microbial contamination. This opinion is strengthened by the huge amount of water wastage associated with open water systems. Most of this wasted water is presumably displaced onto the bedding, regardless of substrate, that could be a source of the bacterial contamination of the bedding, as well as contribute to increased NH_3_ levels. The combination of environmental contamination, increased lameness and mortality, and the vastly increased water waste shows the use of pin-metered water lines is a preferable alternative for ducks housed in US management systems.

## SUPPLEMENTARY DATA

**Figure S1.** Key for the frequency histogram in Figure 5C.
